# Fournier's Gangrene and Pneumothorax Secondary to Nontraumatic Duodenal Perforation

**DOI:** 10.1155/cris/3027477

**Published:** 2025-05-22

**Authors:** Ayman Shehadeh, Amer Mansoor, Jordan Bray, Waed Atallah, Jason Mikhail, Richard Spinale, Angad Pordal

**Affiliations:** Department of Surgery, Garden City Hospital, Garden City, Michigan, USA

**Keywords:** case report, Fournier's gangrene, NSTI, peptic ulcer disease, perforation, pneumothorax

## Abstract

Peptic ulcer disease (PUD) results from erosion and ulceration of the upper digestive tract mucosa. Clinical presentations can vary from asymptomatic to severe complications such as perforation, strictures, or bleeding. Perforation can release enteric contents and gas into the abdomen, leading to intra-abdominal sepsis, requiring surgical intervention for source control and repair. We present a case of a 69-year-old male who developed both Fournier's gangrene and a right-sided pneumothorax secondary to a nontraumatic perforated duodenal ulcer. The patient underwent an emergent thoracostomy, laparotomy with Graham omentoplasty, and extensive debridement with successful outcome. While rare complications like pneumothorax and necrotizing soft tissue infections have been documented, their simultaneous occurrence from a nontraumatic ulcer perforation is unprecedented in literature. Previous reports suggest enteric contents can traverse retroperitoneal fascial planes and peritoneal defects to reach distant anatomical sites as a possible mechanism for these complications. This case highlights the potential for atypical presentations of PUD and the importance of comprehensive evaluation, early recognition, and prompt surgical intervention.

## 1. Introduction

Peptic ulcer disease (PUD) involves erosion and ulceration of the upper digestive tract mucosa, often in the stomach or duodenum, with clinical presentations ranging from asymptomatic to severe complications like gastrointestinal bleeding, perforation, and gastric outlet obstruction [1, 2]. The lifetime prevalence of PUD has decreased with the widespread use of antisecretory drugs, such as proton pump inhibitors, and is currently estimated at 5%–10% [3, 4]. While the overall incidence is declining, complications such as bleeding, perforation, and obstruction still account for nearly 150,000 hospitalizations annually in the United States, with ulcer perforation occurring in approximately 4–14 cases per 100,000 individuals [5, 6].

PUD typically results from imbalances between digestive-promoting factors and protective mechanisms within the gastrointestinal mucosa [1–4]. After a meal, gastric acid is secreted to aid digestion, which then stimulates the release of somatostatin to inhibit further acid secretion through a negative feedback loop [1–4]. Additional mucosal defenses, such as bicarbonate secretion, adequate blood flow, and cellular repair, are regulated by prostaglandins and nitric oxide. Excessive acid production can lead to mucosal erosion, making these protective factors crucial for maintaining mucosal integrity [1–4]. Contributors to PUD such as *H. pylori* infections or excessive NSAID use can disrupt this balance by reducing somatostatin secretion, increasing gastric acid levels, or inhibiting COX-1 derived prostaglandins. Such disruptions weaken mucosal integrity, potentially leading to tissue breakdown and increased exposure of the gastrointestinal epithelium to acid, thereby initiating the ulcerative process [1–4].

This can further progress to the serosal layer and potentially lead to perforation if the serosa is breached [1, 7]. Ulcer perforation is considered a surgical emergency due to the high risk of severe, potentially life-threatening complications and is associated with short-term mortality and morbidity in up to 30%– 50% of patients, respectively [3, 8]. This condition can lead to the leakage of gastrointestinal contents into the peritoneal cavity, resulting in severe peritonitis and subsequent sepsis due to the uncontrolled release of bowel contents into the abdominal cavity. While duodenal perforation can lead to sepsis-related complications and septic shock, it is uncommon for the leakage of enteric contents to cause necrotizing soft tissue infection. Similarly, nontraumatic perforation rarely results in pneumothorax.

In the following case, we describe a rare and atypical set of complications arising from a duodenal ulcer perforation. Specifically, the patient developed Fournier's gangrene, a rapidly progressive necrotizing infection of the genital, perineal, or perianal regions. In addition to this, the patient also simultaneously developed a right-sided pneumothorax.

## 2. Case Report

We present the case of a 69-year-old male who presented to the Emergency Department secondary to a 2-week history of abdominal pain, scrotal pain, and a 2-day history of black tarry stools. The patient's past medical history was unremarkable and the patient denied any recent traumatic events.

**Figure 1:**
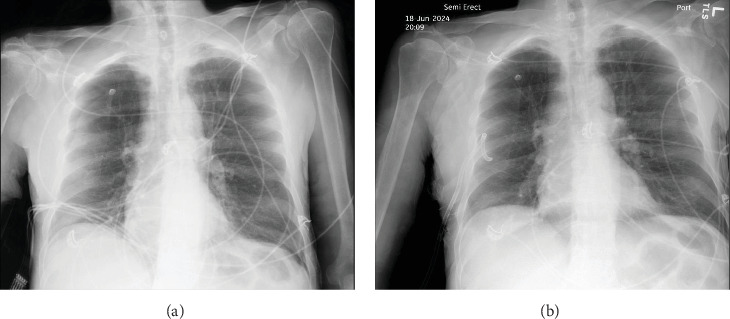
(a) Chest X-ray on arrival demonstrating a right-sided pneumothorax. (b) Chest X-ray demonstrating reexpansion of lung after placement of thoracostomy.

Upon arrival, the patient began endorsing new onset shortness of breath and chest pain and became increasingly agitated. The patient was normotensive and afebrile, yet tachycardic with a heart rate of 142. He was also found to be markedly tachypneic and hypoxic, with an oxygen saturation of 88% despite administration of supplemental oxygen through a nonrebreather mask. A chest X-ray was obtained and the patient was found to have a right-sided pneumothorax with greater than 50% collapse of the lung (Figure 1a). A right-sided tube thoracostomy was performed with evacuation of a large amount of air; however, the patient remained tachycardic and tachypneic despite a follow-up chest X-ray demonstrating a resolving pneumothorax (Figure 1b).

Physical examination demonstrated a distended and diffusely tender abdomen, bilateral testicular swelling with overlying erythema, and a fluctuant, crepitant, erythematous lesion on the medial aspect of the left gluteal region.

The laboratory results indicated leukocytosis with a white blood cell count of 26.0, a hemoglobin level of 14.5, sodium level of 130, creatinine level of 1.37, glucose level of 367, and a C-reactive protein of 190.0. The patient also exhibited elevated lactic acidosis with a lactate level of 4.3.

**Figure 2:**
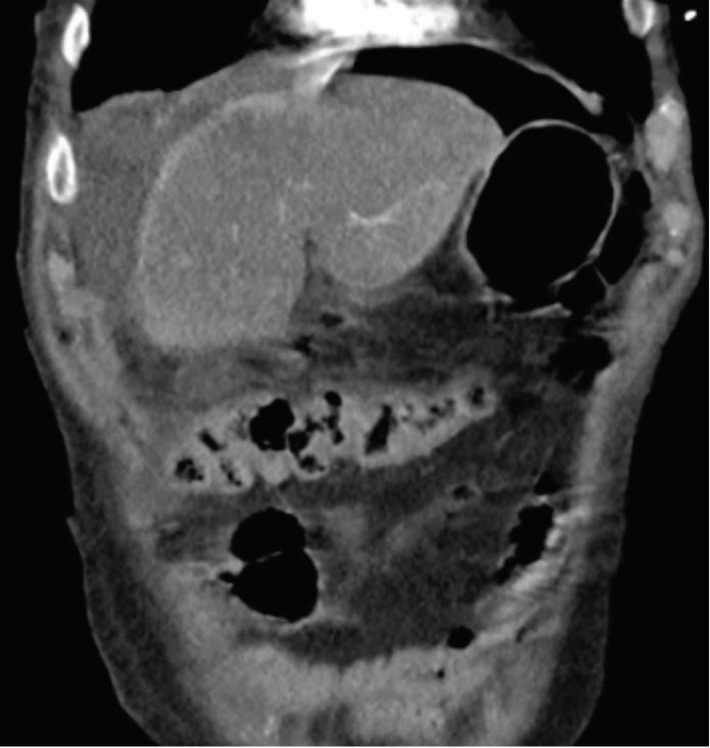
Coronal CT image obtained on arrival demonstrates pneumoperitoneum and intra-abdominal free fluid, consistent with duodenal perforation.

**Figure 3:**
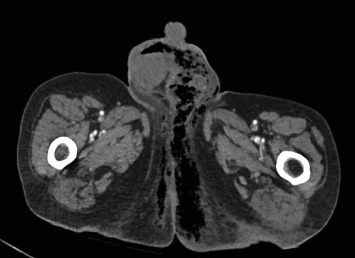
Axial CT image obtained on arrival reveals the presence of air loculi within the perineum and scrotum, consistent with necrotizing soft tissue infection.

To further assess the extent of the patient's suspected soft tissue infection, a CT scan of the chest, abdomen, and pelvis was performed, which demonstrated evidence of free intraperitoneal air and fluid, indicating a hollow viscus perforation near the pyloric antrum or proximal duodenum (Figure 2). Additionally, the scan showed loculi of air and fluid within the soft tissues of the left inguinal region and concerning for Fournier's gangrene (Figure 3). The scan also demonstrated an improvement in the right pneumothorax following chest tube placement.

Afterwards, the patient was emergently taken to the operating room for an exploratory laparotomy and excisional wound debridement of Fournier's gangrene. Endotracheal intubation and general anesthesia were administered. The patient was placed in lithotomy position and his abdomen along with the perineum was prepped and draped. Attention was initially directed to the perineum. An area of skin necrosis and crepitus was appreciated over the patients left gluteal region and perineum. An elliptical incision resulted in an immediate expression of purulent drainage consistent with necrotizing fasciitis. The wound tracked superiorly to the level of the pubic symphysis and posteriorly into the gluteal muscles with a degree of perirectal fat involvement and no additional rectal involvement. Involvement of the skin of the scrotum and peri-testicular tissues were seen which were debrided. The area was then packed with wet-to-dry dressing.

**Figure 4:**
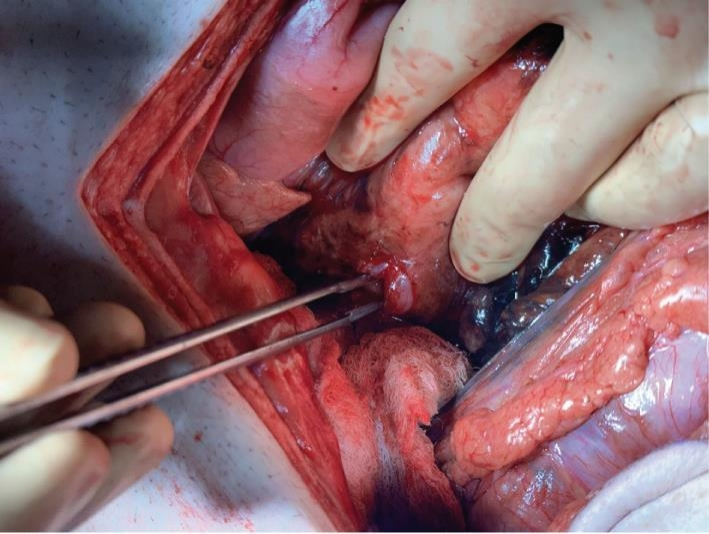
Intraoperative finding of a 1.2 cm perforation along the anterior aspect of the first section of the duodenum, located just beyond the gastric pylorus.

Attention was then directed to the abdomen. A midline laparotomy incision expressed a large amount of free air from the abdomen with copious amounts of bilious succus. Upon further exploration, an anterior perforation was identified in the first part of the duodenum, just distal to the gastric pylorus, measuring approximately 1.2 cm (Figure 4). The tissue along the edge of the perforation was friable and appeared necrotic and was subsequently debrided. Additional tissue along the perforation was excised and sent for pathology to rule out malignancy. Due to the size of the perforation and location, Graham patch omentoplasty was performed approximating the omentum over top of the duodenal perforation. The abdomen was examined closely for any additional injuries and was irrigated with copious amounts of warm saline. Due to the patient's septic shock and need for vasopressor support, a temporary abdominal wound closure was placed, and the patient was transferred to the ICU.

**Figure 5:**
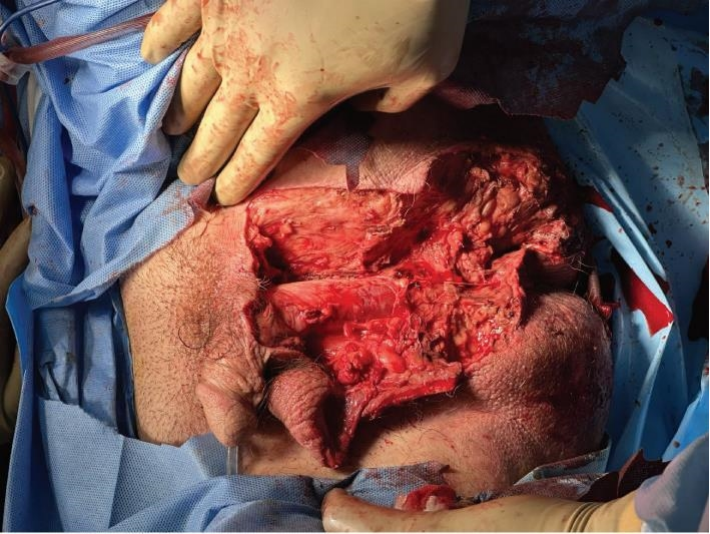
Image of perineum after extensive debridement of Fournier's gangrene.

The patient returned to the operating room approximately 48 h later for a second look. Upon evaluation of the perineum, there was demarcation of necrotic scrotum involving the muscle, fascial layers, and the tunica vaginalis of the testicles. The scrotum was then fully excised and the areas of necrosis within the tunica vaginalis were debrided (Figure 5). The testicles were examined closely and appeared viable. To protect the testicles, a transposition to the superficial inguinal pouch of the right and left leg was performed. The abdomen was then re-assessed closely. There was no reaccumulation of bilious succus or evidence of additional bowel injury. The Graham omentoplasty repair appeared successful. The decision was made, however, to create a diverting sigmoid colostomy given the location of the perineal wound with perirectal involvement. Jackson–Pratt (JP) drains were placed near the right and left paracolic gutters. The abdomen was successfully closed. At the conclusion of the case, the patient remained intubated and returned to the ICU. Patient was extubated the following morning.

**Figure 6:**
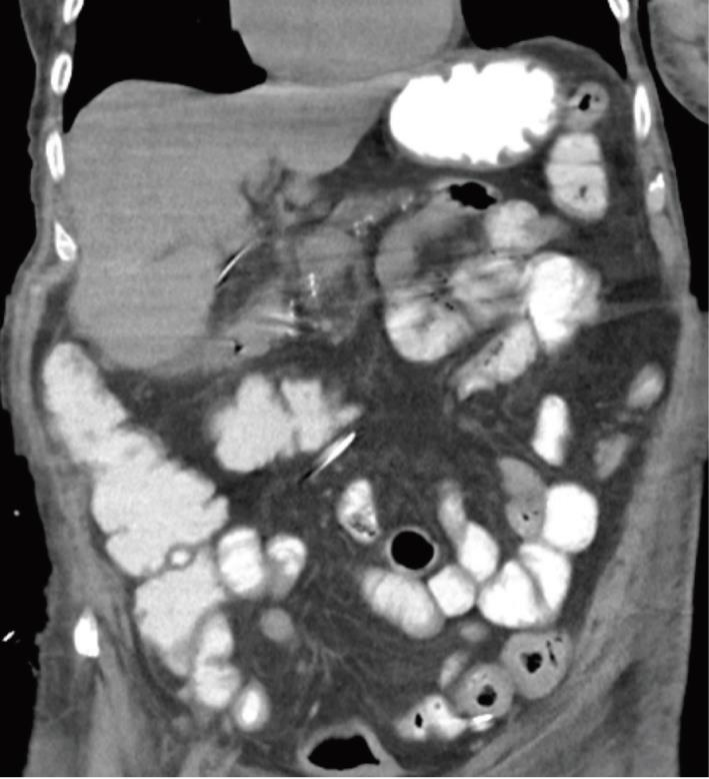
Oral contrast-enhanced CT on postoperative Day 7 demonstrating no contrast extravasation, indicative of the absence of an anastomotic or perforation-related leak.

Postoperatively, the patient had an uneventful course. Broad spectrum antibiotic and antifungal coverage along with aggressive fluid resuscitation was performed resulting in resolution of septic shock. Antibiotic therapy was tailored to the patient's blood cultures which resulted in gram-positive coagulase-negative staphylococcus. Daily dressing changes of the patient's perineal wound was performed with we-to-dry Dakin's-soaked dressings. The wound was inspected with daily dressing changes without evidence of further purulence or necrosis. Patient was eventually transitioned to negative pressure wound therapy. On postoperative Day 7 after the Graham patch omentoplasty, the patient underwent gastrografin evaluation of his Graham patch omentoplasty which showed no leak (Figure 6). He had a return of bowel function with adequate stool and gas per ostomy. JP drains remained non-bilious and serous. Patient's diet was slowly advanced which he tolerated. He was eventually discharged to a long-term care facility. JP drains were removed prior to discharge. He was seen in the clinic at 3 months from his index operation and was found to have improvement in the size of his wound with healthy granulation tissue seen throughout.

Pathology of the duodenal perforation demonstrated surface denudation and acute inflammation consistent with an ulcer without evidence of malignancy. Pathology of scrotal tissue demonstrated extensive ulceration and necrosis.

## 3. Discussion

This case describes a rare and atypical set of complications following a duodenal ulcer perforation, including Fournier's gangrene, a life-threatening infection of the genital and perineal regions and a right-sided pneumothorax, both occurring without any prior traumatic events. Although these conditions can occur simultaneously due to unrelated pathologies, previous reports suggest a substantial pneumoperitoneum resulting from a perforated viscus can contribute to the development of pneumothorax. For example, Marwan et al. [9] describes a case of a pneumothorax secondary to an iatrogenic perforation during a colonoscopy. In this report, the author suggests that air from pneumatosis coli can dissect through the colonic wall into the retroperitoneum and spread along fascial planes into the pleural and mediastinal spaces leading to a pneumothorax. Another mechanism discussed is the possibility of air passing through diaphragmatic openings from the peritoneal cavity into the pleural space due to a pressure gradient. Benichou et al. [10] describe a case where a patient who underwent abdominal surgery developed both pneumoperitoneum and right-sided pneumothorax without diaphragmatic trauma, implying a link between pneumothorax and intestinal perforation with pneumoperitoneum, even in the absence of visible diaphragmatic or mediastinal defects. Several other mechanisms have been proposed for how extraluminal air from an intestinal perforation can enter the thoracic cavity and cause pneumothorax in the absence of diaphragmatic defects. Utzig et al. [11] describes a case in which a pneumothorax developed secondary to a perforated duodenal ulcer, proposing that free intraperitoneal air under tension may diffuse through inflamed peritoneal membrane and into the pleural cavity. In another report, Ball et al. [12] describes a case of a pneumothorax secondary to an intestinal perforation and suggests that retroperitoneal free air may gain access to the pleural cavity by dissecting through fascial planes.

In the case we present here, the patient's presentation was further complicated with Fournier's gangrene, a severe subtype of necrotizing soft-tissue infections (NSTIs) that primarily affects the perineal, anal, and genital regions [13, 14]. NSTIs typically spread along fascial planes, initially sparing the skin but later leading to ischemia as bacteria invade cutaneous blood vessels [15]. This condition can be initiated by the translocation or perforation of the bowel, leading to the release of intestinal flora, leading to a polymicrobial infection predominantly involving gram-negative bacilli and anaerobic bacteria [8]. In this case, however, blood cultures grew for gram-positive coagulase-negative staphylococcus, which is not consistent with the typical microbial infections expected from a duodenal perforation.

Early intervention is crucial, as mortality rates can significantly increase with a delayed diagnosis and can rise to 40%–50% [14]. In contrast, when diagnosed and treated promptly, the mortality rate can be as low as 10%–15% [16]. When NSTI is suspected, exploration and wide surgical debridement are essential to remove necrotic tissue and obtain source control, follow by administering intravenous fluids and broad-spectrum antibiotics until the patient stabilizes [17].

We postulate that the leakage of succus from the duodenal perforation into the abdominal cavity likely tracked retroperitoneally through fascial planes or through various peritoneal defects, such as a patent processus vaginalis, reaching the pelvic region and then the scrotum via the inguinal canal, ultimately leading to Fournier's Gangrene. While it is possible that the patient experienced two separate etiologies accounting for both the duodenal perforation and the necrotizing infection, the substantial amount of succus found intraperitoneally, particularly within the pelvis as a result of the perforation, suggests a connection between the two pathologies. Extensive debridement of the perianal region and the entirety of the scrotum was necessary due to the extent of necrotic tissue. The testicles, however, were spared likely due to the gonadal artery blood supply that arises from directly from the aorta and is independent from the affected region as described in previous literature [18, 19]

A delayed diagnosis and treatment of a perforated duodenal ulcer can lead to a cascade of complications, potentially culminating in septic shock or other severe, life-threatening conditions. Necrotizing soft tissue infections and pneumothorax are two rare and unexpected complications of a perforated duodenal ulcer, each associated with significant morbidity and mortality. Despite these challenges, this patient was successfully managed with surgical intervention and was ultimately discharged from the hospital with a successful outcome. Overall, this case underscores the potential for atypical presentations of duodenal perforation and highlights the importance of thorough evaluation, prompt recognition, and urgent surgical management of perforated PUD.

## Data Availability

The data that support the findings of this study are available upon request from the corresponding author. The data are not publicly available due to privacy or ethical restrictions.

## References

[1] Stern E., Sugumar K., Journey J. D. (2023). *Peptic Ulcer Perforated*.

[2] Tuerk E., Doss S., Polsley K. (2023). Peptic Ulcer Disease. *Primary Care: Clinics in Office Practice*.

[3] Lanas A., Chan F. K. L. (2017). Peptic Ulcer Disease. *The Lancet*.

[4] Kempenich J. W., Sirinek K. R. (2018). Acid Peptic Disease. *Surgical Clinics of North America*.

[5] Feinstein L. B., Holman R. C., Christensen K. L. Y., Steiner C. A., Swerdlow D. L. (2010). Trends in Hospitalizations for Peptic Ulcer Disease, United States, 1998–2005. *Emerging Infectious Diseases*.

[6] Xie X., Ren K., Zhou Z., Dang C., Zhang H. (2022). The Global, Regional and National Burden of Peptic Ulcer Disease From 1990 to 2019: A Population-Based Study. *BMC Gastroenterology*.

[7] Søreide K., Thorsen K., Harrison E. M. (2015). Perforated Peptic Ulcer. *The Lancet*.

[8] Møller M. H., Adamsen S., Thomsen R. W., Møller A. M. (2011). Multicentre Trial of a Perioperative Protocol to Reduce Mortality in Patients With Peptic Ulcer Perforation. *British Journal of Surgery*.

[9] Marwan K., Farmer K. C., Varley C., Chapple K. S. (2007). Pneumothorax, Pneumomediastinum, Pneumoperitoneum, Pneumoretroperitoneum and Subcutaneous Emphysema Following Diagnostic Colonoscopy. *Annals of The Royal College of Surgeons of England*.

[10] Benichou J., Benichou J., Owen C. (2021). Right-Sided Pneumothorax Associated With Pneumoperitoneum Subsequent to Transverse Colon Perforation: Case Report. *Journal of Surgical Oncology*.

[11] Utzig M. J., Foitzik T., Buhr H. J., Schneider P. (2002). Pneumomediastinum und Pneumothorax als Manifestation eines Ulcus duodeni—Fallbeschreibung [Duodenal Ulcer Presenting as Pneumomediastinum and Pneumothorax—Case Report]. *Zentralblatt für Chirurgie*.

[12] Ball C. G., Kirkpatrick A. W., Mackenzie S. (2006). Tension Pneumothorax Secondary to Colonic Perforation During Diagnostic Colonoscopy: Report of a Case. *Surgery Today*.

[13] Tang L.-M., Su Y.-J., Lai Y.-C. (2015). The Evaluation of Microbiology and Prognosis of Fournier’s Gangrene in Past 5 Years. *SpringerPlus*.

[14] Oguz A., Gümüs M., Turkoglu A. (2015). Fournier’s Gangrene: A Summary of 10 Years of Clinical Experience. *International Surgery*.

[15] Corcoran A. T., Smaldone M. C., Gibbons E. P., Walsh T. J., Davies B. J. (2008). Validation of the Fournier’s Gangrene Severity Index in a Large Contemporary Series. *Journal of Urology*.

[16] Parry N. (2015). Fournier Gangrene. *Clinical Case Reports*.

[17] Shyam D. C., Rapsang A. G. (2013). Fournier’s Gangrene. *The Surgeon*.

[18] Anzai A. K. (1995). Fournier’s Gangrene: A Urologic Emergency. *American Family Physician*.

[19] Koukouras D., Kallidonis P., Panagopoulos C. (2011). Fournier’s Gangrene, A Urologic and Surgical Emergency: Presentation of a Multi-Institutional Experience With 45 Cases. *Urologia Internationalis*.

